# Effect of *Acacia catechu* (L.f.) Willd. on Oxidative Stress with Possible Implications in Alleviating Selected Cognitive Disorders

**DOI:** 10.1371/journal.pone.0150574

**Published:** 2016-03-07

**Authors:** Manas Ranjan Saha, Priyankar Dey, Sainiara Begum, Bratati De, Tapas Kr. Chaudhuri, Dilip De Sarker, Abhaya Prasad Das, Arnab Sen

**Affiliations:** 1 Molecular Cytogenetics Laboratory, Department of Botany, University of North Bengal, Siliguri, 734013, India; 2 Cellular Immunology Laboratory, Department of Zoology, University of North Bengal, Siliguri, 734013, India; 3 Phytochemistry and Pharmacognosy Research Laboratory, Department of Botany, University of Calcutta, 35 Ballygunge Circular Road, Kolkata, 700019, India; 4 Department of Botany, Raiganj University, Raiganj, 733134, India; 5 Taxonomy and Envioronmental Biology Laboratory, Department of Botany, University of North Bengal, Siliguri, 734013, India; Indian Institute of Integrative Medicine, INDIA

## Abstract

In human body, several categories of degenerative processes are largely determined by free radicals originating in cell. Free radicals are also known to have correlated with a variety of cognitive disorders (CDs) resulting in neuronal injury and eventually to death. Alzheimer’s disease (AD) and Parkinson's disease (PD) are such kind of killer CDs that occur due to dysfunction of cholinergic and dopaminergic neurons. Plant parts of *Ginkgo biloba*, *Bacopa monnieri* etc. are being used for the treatment of cognitive disorders in several countries. The present study was aimed to explore the detailed antioxidant and anti-cholinesterase activity of *Acaciacatechu* leaf (ACL) over CDs. Gas chromatography-Mass spectroscopy (GC-MS) analysis and Nuclear Magnetic Resonance (NMR) were employed to identify the bioactive components present in ACL. Furthermore, the extract was evaluated to check the cytotoxic effects of ACL on normal cells. Amongst several antioxidant assays, DPPH assay, hydroxyl radical, nitric oxide radical and hypochlorous acid inhibitory activities were found to be greater in ACL than that of the respective standards while other assays exhibited a moderate or at per inhibitory activity with standards. Total phenolic and flavonoid content were also found to be present in decent amount. In addition, we found, a greater acetylcholinesterase (AChE) inhibitory activity of ACL when compared to other medicinally important plants, indicating its positive effect over CDs. Forty one bioactive components were explored through GC-MS. Of these, gallic acid, epicatechin, catechin, isoquercitrin etc. were found, which are potent antioxidant and a few of them have anti-neurodegenerative properties. Eventually, ACL was found to be nontoxic and safer to consume. Further studies with animal or human model however, would determine its efficacy as a potential anti-schizophrenic drug.

## Introduction

Generation of highly Reactive Oxygen Species (ROS) is an integral feature of a normal cell [1]. Of late, knowledge of free radicals and ROS has brought about a revolution in the domain of health and disease management [2]. The free radicals, including superoxide anion, hydrogen peroxide, hydroxyl radicals, nitric oxide, peroxynitrite etc. have potential to initiate degenerative processes in human body and react with important cellular components like DNA, cell membranes and proteins [3]. In fact, there is a proper balance between ROS and enzymatic defense system i.e. oxidant, antioxidant balance in a normal cell. This balance is shifted when antioxidant level is reduced, which is known as oxidative stress causing serious cell damage leading to atheroscleorosis, arthritis, cancer and a variety of cognitive disorders (CDs) like Alzheimer’s disease (AD) and Parkinson’s disease (PD) etc [4].

Cognitive disorders are those disorders that affect the brain's ability to remember and process information [5]. Beside AD and PD, cognitive disorders include dementia, amnesia and hallucination. AD is characterized by loss or decline of memory, language deterioration, poor judgment and cognitive impairment and mainly found in elderly persons [6]. AD is believed to be linked to a deficiency in the brain neurotransmitter, acetylcholine. Inhibition of acetylcholinesterase (AChE) is important for the systemic treatment of AD [6]. PD, the second most common neurodegenerative disorder after AD, is characterized by resting tremor, bradykinesia, muscular rigidity, and postural imbalance occurring due to progressive death of substantia nigral cells leading to dysfunction of dopaminergic neurons [7]. Moreover, ROS are continuously generated in brain leading to a progressive accumulation of cellular damage which is correlated with AD and PD [7].

Donepezil, tacrine, rivastigmine, galanthamine, levodopa, apomorphine etc. are some of the commercial drugs used for the treatment of cognitive disorders including AD and PD. However, they show several adverse effects in human including insomnia, anorexia, diarrhea, fatigue, nausea, gastrointestinal disorders and cardiovascular disorders [6]. The plant products on the other hand are long been used in curing several cognitive disorders without having much side effects. *Ginkgo biloba*, *Catharanthus roseus*, *Bacopa monnieri*, *Acorus calamus*, *Centella asiatica* etc. are some of the plants whose extracts are routinely used in herbal formulation as remedy for AD [8–10]. What's more, herbal products have antioxidant properties which may help fighting against reactive oxygen species (ROS) [2, 3, 11, 12].

*Acacia catechu* (L.f.) Willd. or Khair (AC), belonging to the family Mimosaceae is used in most of the herbal preparations of ayurveda in India. Traditionally, Khair is beneficial against several gastrointestinal and stomach related ailments, and leprosy [13, 14]. Some antioxidative properties of AC heartwood extract have also been reported [15, 16]. However, no significant work has been done with *Acacia catechu* leaf (ACL) extract; especially so far there is no report of anti-acetylcholinesterase, anti-schizophrenic or anti-cognitive disorder properties of ACL extract. A preliminary study exhibited that *A*. *catechu*-catechin helps to improve behavioral patterns in animals [17]. However, the mechanism is still unknown. Therefore, in the present study, we attempted probably for the first time, to work out the possible mechanism of activity of ACL extract for neuroprotection and prevention of CDs. To evaluate the free radical scavenging level of ACL extract, a detailed antioxidant profiling has been done which is directly associated with CDs like Parkinson’s, Alzheimer’s, trauma, seizures etc [6]. Besides, we also evaluated the cytotoxicity of ACL to access the safety of its consumption. Emphasis was also given on those chemical compounds which might have potent role in counteracting oxidative damage.

## Materials and Method

### Plant material

The plant, *A*. *catechu* (L.f.) Willd. was authenticated by the Taxonomists of Department of Botany, University of North Bengal, India and a voucher specimen (No.- NBU/UD/1039) was deposited at the herbaria of the same Department. The fresh leaves of the *Acacia catechu* were collected during the month of September 2013.

### Ethics statement

The plant sample was collected from the medicinal plant garden of Department of Botany, University of North Bengal, West Bengal, India. The garden is not within a National Park/Reserve Forest/Govt. protected area, therefore, only verbal but formal permission from the respective Department was obtained before collection and the study did not involve any endangered or protected species.

All the experiments using animals were reviewed and approved by the Animal Ethical Committee of the University of North Bengal (Permit No. 840/ac/04/CPCSEA, Committee for the Purpose of Control and Supervision on Experiments on Animals) and performed in accordance with the legislation for the protection of animals used for scientific purposes.

### Chemicals and reagents

Chemicals and reagents in the present study were of analytical grade and purchased either from HiMedia Laboratories Pvt. Ltd., Mumbai, India or Merck, Mumbai, India, or Sigma-Aldrich, USA. The EZcount^™^ MTT (3-(4,5-Dimethylthiazol-2-yl)-2,5-diphenyltetrazolium bromide) Cell Assay Kit was procured from HiMedia Laboratories Pvt. Ltd. (Mumbai, India).

### Preparation of extract

Fresh leaves of AC were air-dried for 2–3 weeks and then pulverized to fine powder by electric grinder. Exhaustive extraction was performed in Soxhlet apparatus for 11 h using ethanol as a solvent. The extract was then concentrated under reduced vacuum pressure at 40°C in a rotary vacuum evaporator (Buchi Rotavapor R-3, Switzerland). The concentrated extracts were further lyophilized using Eyela Freeze Dryer (FDU-506, USA). Finally, the lyophilized extract was stored in sterile container and placed in -20°C until further use.

### *In-vitro* antioxidant assays

In order to determine the ability of ACL extract to serve as antioxidant against free radicals or ROS, a series of antioxidant assays were performed first.

#### DPPH (2, 2-diphenyl-1-picrylhydrazyl) radical scavenging assay

Free radical scavenging activity through DPPH assay was performed as per Chewet al. [18] with a brief modification. Various concentrations of plant extracts (0–100 μg/ml) were prepared and mixed properly with freshly prepared DPPH solution (1mM; diluted in 95% methanol) and kept in dark. Optical density (OD) was measured after 30 minutes of reaction at 517 nm using UV-Vis Spectrophotometer (Thermo UV1, Thermo Electron Corporation, England, UK). Ascorbic acid was taken as standard. The percent radical scavenging activity was calculated using Equation I:

$$\text{Percentage}\;\text{of}\;\text{scavenging}\;\text{DPPH}=\frac{A_{0}-A_{1}}{A_{0}}\;\times 100$$

Where, A_0_ = absorbance of the control and A_1_ = absorbance in the presence of samples and standard.

#### Reducing power assay

The Fe^3+^-reducing power of ACL extract was evaluated by the method of Oyaizu et al. [19] with slight alterations. Different concentrations (0–64 μg/ml) of plant extract (0.5 ml) were mixed with 0.2 M of phosphate buffer (0.5 ml, pH 6.6) and 0.1%of potassium hexacyanoferrate (0.5 ml), followed by incubation at 50°C for 20 min. in a water bath. After incubation, 0.5 ml of TCA (10%) was added to the mixture to terminate the reaction. The upper portion of the reaction mixture (1 ml) was then transferred to another tube and mixed with 1 ml of distilled water followed by 0.1 ml of FeCl_3_ solution (0.01%). The mixture was left for 10 min at room temperature and the absorbance was measured at 700 nm against an appropriate blank solution. Butylated hydroxytoluene (BHT) was used as standard.

#### Hydroxyl radical scavenging assay

Hydroxyl radical scavenging assay of ACL was carried out on the basis of Fenton reaction [20] with a few changes. A reaction mixture was prepared containing 2-deoxy-2-ribose (2.8 mM), monopotassium phosphate-potassium hydroxide buffer (KH_2_PO_4_-KOH; 20 mM; pH 7.4), ferric chloride (FeCl_3_; 100 μM), ethylenediaminetetraacetic acid (EDTA; 100 μM), hydrogen peroxide (H_2_O_2_; 1.0 mM), ascorbic acid (100 μM) and various concentrations of extracts (0–200 μg/ml) up to a final volume of 1 ml and the reaction mixture was left for 1 h incubation at 37°C. Following incubation, 0.5 ml of incubated mixture was taken into another tube and mixed with 1 ml of tricarboxylic acid (TCA; 2.8%) and 1 ml of aqueous thiobarbituric acid (TBA; 1%). The final mixture was incubated at 90°C for 15 min then cooled down to room temperature and the absorbance was measured at 532 nm against a blank solution. Mannitol was used as positive control. Percentage of inhibition was evaluated following Eq I.

#### Superoxide radical scavenging assay

This assay was performed by the reduction of nitro blue tetrazolium (NBT) as described by Fontana et al. [21] with brief modifications. Generally, the nonenzymatic phenazine methosulfate-nicotinamide adenine dinucleotide (PMS/NADH) system generates superoxide radicals, which reduce NBT to a purple formazan. The reaction mixture (1 ml) contained phosphate buffer (20 mM, pH 7.4), NADH (73 μM), NBT (50 μM), PMS (15 μM) and various concentrations (0–100 μg/ml) of plant extract. After incubation for 5 min at ambient temperature, the absorbance at 562 nm was measured against an appropriate blank to determine the quantity of formazan generated. Quercetin was used as standard.

#### Singlet oxygen scavenging assay

The production of singlet oxygen (_1_O^2^) was determined by monitoring the bleaching of N, N-dimethyl-4-nitrosoaniline (RNO) using the method of Pedraza-Chaverrí et al. [22] with minor modifications. The reaction mixture contained 45 mM phosphate buffer (pH 7.1), 50 mM NaOCl, 50 mM H_2_O_2_, 50 mM L-histidine, 10 μM RNO and various concentrations (0–200 μg/ml) of plant extract to make final volume of 2 ml. The mixture was then incubated for 40 min at 30°C and decrease in the absorbance of RNO was measured at 440 nm. Lipoic acid was used as a reference compound. Singlet oxygen scavenging activity was calculated using the Eq I.

#### Nitric oxide radical scavenging assay

The nitric oxide radical quenching activity was performed following the GriessI-llosvoy reaction [23] with minor modifications. Briefly, phosphate buffered saline (pH 7.4), sodium nitroprusside (SNP; 10 mM) and various concentrations of ACL (0–100μg/ml) were mixed to make final volume of 3 ml. After incubation for 150 minutes at 25°C, 1 ml of sulfanilamide (0.33%; diluted in 20% of glacial acetic acid) was added to 0.5 ml of the pre-incubated reaction mixture and left for 5 min. Following the incubation, 1 ml of N-(1-Naphthyl)ethylenediamine dihydrochloride (NED; 0.1%) was added and incubated for 30 min at 25°C to develop the color. The absorbance was measured spectrophotometrically at 540 nm against blank sample. Curcumin was used as standard. The percentage inhibition was calculated using Eq I.

#### Peroxynitrite scavenging assay

Peroxynitrite (ONOO^-^) was prepared following the method of Beckman et al. [24]. Briefly, an acidic solution (0.6M HCl) was prepared mixing with 5 ml of H_2_O_2_ (0.7 M) and 5 ml of KNO_2_ (0.6 M) on an ice bath for 1min. Then 5 ml of ice-cold NaOH (1.2 M) was added to the mixture. Excess H_2_O_2_ was removed by the treatment with granular MnO_2_ prewashed with NaOH (1.2 M) and the reaction mixture was left overnight at -20°C. Finally, peroxynitrite solution was collected from the top of the frozen mixture and the concentration was measured spectrophotometrically at 302 nm (ε = 1670 M-1 cm-1).

Peroxynitrite scavenging activity was measured by Evans Blue bleaching assay. The assay was carried out as per the method of Bailly et al. [25] with a slight modification. A reaction mixture was prepared containing phosphate buffer (50 mM; pH 7.4), DTPA (0.1 mM), NaCl (90 mM), KCl (5 mM), 12.5 μM of Evans Blue, various doses of plant extract (0–200 μg/ml) and 1 mM of peroxynitrite in a final volume of 1 ml. After incubation at 25°C for 30 min the absorbance was measured at 611 nm. The scavenging percentage of ONOO^-^ was calculated by comparing the results of the test and blank samples. Gallic acid was used as the reference compound.

#### Hypochlorous acid scavenging assay

Hypochlorous acid (HOCl) was prepared freshly by mixing 10% (v/v) solution of NaOCl to 6.2 (pH) with 0.6 M H_2_SO_4_ and the concentration was determined by measuring the absorbance at 235 nm using the molar extinction coefficient of 100 M-1 cm-1 as per Aruoma et al. [26] with few changes. A reaction mixture was prepared containing 50 mM phosphate buffer (pH 6.8), catalase (7.2 μM), HOCl (8.4 mM) and plant extract of different concentrations (0–100 μg/ml) into a final volume of 1 ml. The mixture was incubated for 20 min at 25°C and absorbance was measured against an appropriate blank. The quenching activity was accessed by measuring the decrease in absorbance of catalase at 404 nm. Ascorbic acid was used as standard.

#### Iron chelation assay

The ferrous ion chelating activity was carried out as per the method of Haro-Vicenteet al. [27] with slight changes. Various concentrations of ACL (0–200μg/ml) were mixed properly with ferrous sulfate solutions (12.5 μM) in HEPES buffer (20 mM; pH 7.2) followed by the addition of ferrozine (75 μM) to initiate reaction. The reaction mixture was shaken vigorously and incubated for 20 min at room temperature. The absorbance was measured at 562 nm. EDTA was used as positive control.

#### Hydrogen peroxide scavenging assay

The scavenging activity was determined by the method of Long et al. [28] with minor modifications. A mixture was prepared with H_2_O_2_ (50 mM) and various concentrations of plant samples (0–2000μg/ml) and left for 30 min of incubation at room temperature followed by the addition of 90 μl H_2_O_2_, 10 μl of Methanol (HPLC grade) and 0.9 ml of FOX reagent (prepared by mixing 9 volumes of 4.4 mM BHT in HPLC grade methanol with 1 volume of 1 mM xylenol orange and 2.56 mM ammonium ferrous sulfate in 0.25 M H_2_SO_4_). The whole mixture was then vortexed and left for incubation for 30 min. The absorbance was measured at 560 nm. Sodiun pyruvate was used as positive control.

#### Lipid peroxidation inhibition assay

Lipid peroxidation assay was followed by the method of Kizilet al. [29] with a few modifications. Brain homogenate was prepared by centrifuging of Swiss albino mice brain (20 ± 2 g) with phosphate buffer (50 mM; pH 7.4) and potassium chloride (KCl; 120 mM) at 3000 rpm for 10 min. Various concentrations of ACL extracts (0–25 μg/ml) were mixed with the homogenate (100 μl) followed by addition of ferrous sulfate (FeSO_4_; 0.1 mM) and ascorbic acid (0.1 mM) and incubated for 1 h at 37°C. Following incubation, TCA (500 μl; 28%) and TBA (380 μl; 2%) were added in the reaction mixture and then heated at 95°C in water bath for 30 min. Then the mixtures were cooled down to room temperature and centrifuged at 8000 rpm for 2 min. The absorbance of the supernatant was measured at 532 nm. Trolox was used as positive control.

#### Quantification of total phenolic content

The total phenolic content (TPC) was determined using Folin-Ciocalteu reagent [30] with slight changes. Briefly, ACL extract (100 μl) was mixed with 0.75 ml of Folin—Ciocalteu reagent (previously diluted 1000-fold with distilled water) and left for 5 min at room temperature followed by the addition of sodium carbonate (Na_2_CO_3_; 0.06%) to the mixture. After incubation of 90 min at room temperature, the absorbance was measured at 725 nm. The phenolic content was measured against a gallic acid standard curve.

#### Quantification of total flavonoid content

Total flavonoid content was measured using aluminum chloride (AlCl_3_) method [31] with few modifications. Briefly, ACL extract (100μl) was added to 0.3 ml of distilled water followed by addition of NaNO_2_ (5%; 0.03 ml). After 5 min of incubation at room temperature, AlCl_3_ (10%; 0.03 ml) was added and left for 5 min. The reaction mixture was then treated with 0.2 ml of sodium hydroxide (NaOH; 1 mM) and diluted to 1 ml with water. The absorbance was measured at 510 nm. The flavonoid content was determined from a quercetin standard curve.

### Acetylcholinesterase (AChE) inhibition assay

AChE inhibiting activity of ACL was carried out based on Ellmanet al. [32] method with brief modification. Reaction mixture was prepared containing sodium phosphate buffer (0.1 mM), 5, 5'-dithiobis-2-nitrobenzoic acid (DTNB; 0.1mM), various concentrations of plant extracts (0–200 μg/ml) and acetylcholinesterase (2 U/ml) in a 96 well micro plate and incubated for 15 min at 25°C. After incubation, acetylthiocholin iodide (0.05mM) was added as substrate in the reaction mixture and the enzyme activity was measured immediately after 3 min in a Bio-Rad iMark^™^ microplate absorbance reader at 412 nm. Eserine was used as positive control. The percentage of inhibition was calculated in terms of percentage by dividing the difference of sample absorbance from control with control absorbance × 100.

### MTT cell viability assay

The assay was performed to evaluate the cytotoxic properties of ACL extract on murine splenocytes and macrophages. The splenocyte and macrophage cells were collected from Swiss albino mice [33]. Swiss albino mouse was sacrificed under mild ether anesthesia and the spleen was aseptically removed from the body and washed thrice (1000 rpm) with RPMI-1640 and splenocytes suspension was prepared and resuspended in 0.16 M NH_4_Cl (in 0.17 M Tris; pH 7.2) to remove any trace of erythrocytes. After 5 min, the reaction was stopped using chilled RPMI-1640 and the cells were washed as previous. Peritoneal exudate macrophages were collected by washing the mouse peritoneal region with RPMI-1640. Cell suspension (2×10^6^ cells/ml) was prepared with penicillin (50 U/ml), streptomycin (50 U/ml), nystatin (50 U/ml) and fetal bovine serum (FBS, 10%) in RPMI-1640 medium. The cell suspension (100 μl) was added with 100 μl of different concentrations (0–200 μg/ml) of ACL (dissolved in RPMI-1640) to the wells of 96-well plate. The plates were then covered and incubated under 5% CO2 and humidified atmosphere of 90% air at 37°C temperature for 48 h. The cytotoxicity assay was performed according to the manufacturer’s instructions of EZcount^™^ MTT Cell Assay Kit (HiMedia).

### GC-MS analysis

Ethanolic leaf extract of AC was derivatized with 10 μl of methoxyamine hydrochloride (20 mg/ml in Pyridine) and N-methyl-N-trimethylsilyltrifluoroacetamide (MSTFA) with 1% trimethylchlorosilane (TMCS) 2 μl FAME (Fatty Acid Methyl Esters) markers [a mixture of internal Retention Index (RI) markers was prepared using fatty acid methyl esters of C8, C10, C12, C14, C16, C18, C20, C22, C24 and C26 linear chain length, dissolved in chloroform (HPLC) at a concentration of 0.8 mg/ml (C8-C16) and 0.4 mg/ml (C18-C30)] was added [34]. GC-MS analysis was performed following the method of Kind et al. [34] after little modification. HP-5MS capillary column (Agilent J & W; GC Columns (USA) (length 30 m plus Duraguard 10 m, diameter 0.25 mm narrowbore, film 0.25 μm) was used. The analysis was performed under the following oven temperature programme: Injection in sandwich mode with fast plunger speed without viscosity delay or dwell time, oven ramp 60°C (1 minute hold) to 325°C at 10°C/minute, 10 minute hold before cool-down, 37.5 minute run time. The injection temperature was set at 250°C; the MS transfer line at 290°C and the ion source at 230°C. Prior to analysis the method was calibrated with the FAME standards available with the Fiehn GC/MS Metabolomics library (2008) (Agilent ChemStation, Agilent Technologies Inc., Wilmington, USA). Helium was used as the carrier gas at a constant flow rate of 0.723ml / min (carrier linear velocity 31.141 cm/sec). Samples (1 μl) were injected via the split mode (Split ratio 1:5) onto the GC column. Automated mass spectral deconvolution and identification system (AMDIS) was used to deconvolute GC-MS results and to identify chromatographic peaks. Identification of the metabolites was carried out by comparing the fragmentation patterns of the mass spectra, retention times and retention indices with entries of those in Agilent Fiehn Metabolomics library using Agilent retention time locking (RTL) method. Response ratio was calculated as peak area ratios of metabolite and ribitol as internal standard. Relative response ratio (RRR) is normalized response ratio per g crude extract.

### NMR spectroscopic analysis

^1^H and ^13^C NMR spectra of ACL extract were recorded on a 300 MHz Bruker FT-NMR (Avance AV-300) spectrometer. Sample was dissolved in DMSO-d_6_ and the chemical shifts were reported in δ values [35].

### Statistical methods

For the reproducibility, all data were prepared as the mean ± SD of six measurements. Statistical analysis was performed by one-way analysis of variance (ANOVA) with Dunnett’s test using KyPlot version 5.0 beta 15 (32 bit) for windows where p< 0.05 was considered as significant. The graphs were prepared using KyPlot (version 5.0 for windows).

## Results and Discussion

The endogenous free radical forming pathway demonstrates a cascade of diverse free radicals originating from molecular oxygen. Since oxidative stress causes extensive lipid peroxidation and increases the risk of neurodegeneration and subsequent cognitive disorders, exogenous dietary antioxidants proves to be the foremost choice for prevention of such conditions [7].

**Fig 1:**
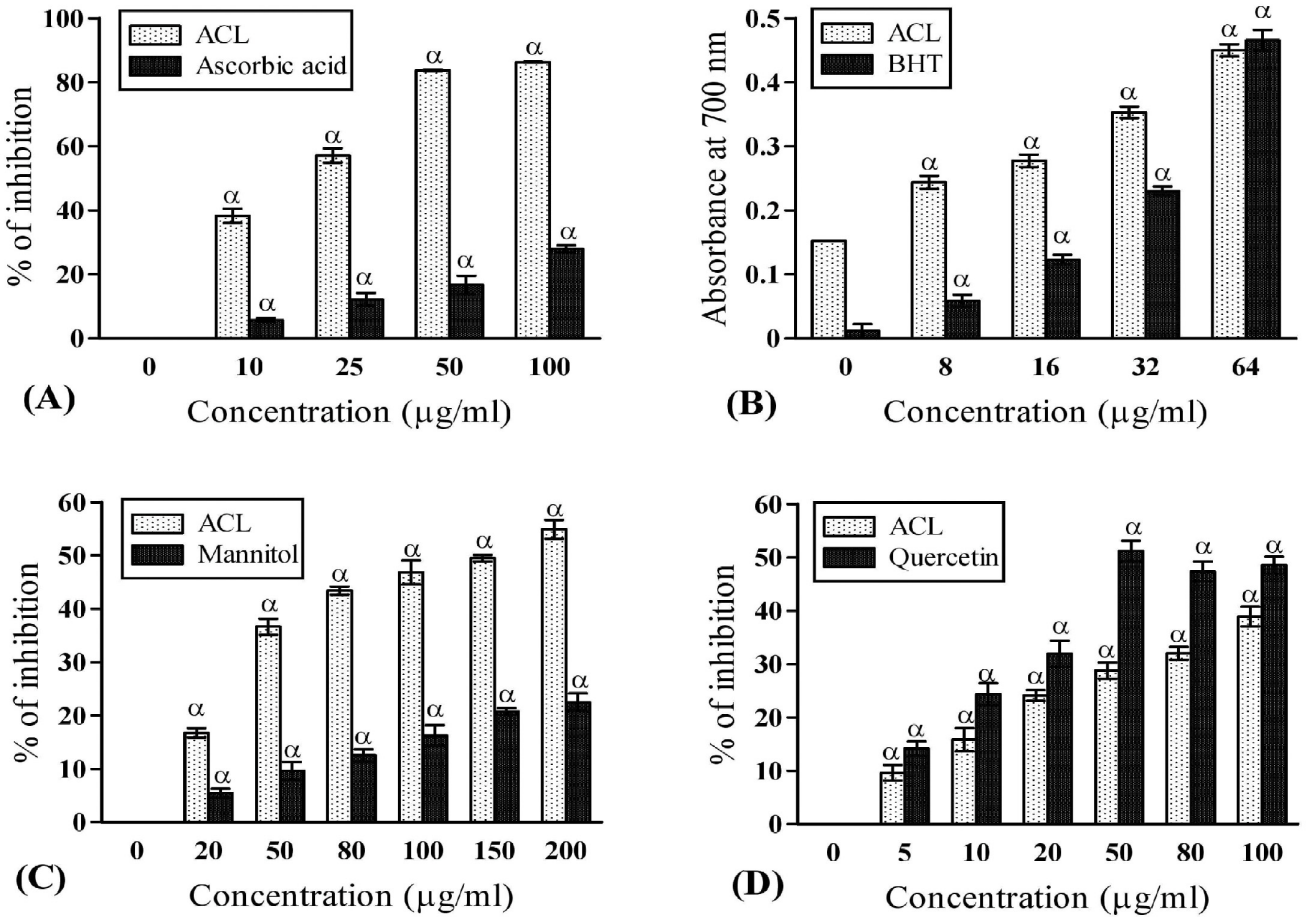
Antioxidant and free-radical scavenging activities of ACL extract. **(A)** DPPH radical scavenging activities of ACL extract and standard ascorbic acid (IC_50_ value: ACL = 15.52±0.46μg/ml and ascorbic acid = 240.10±28.35 μg/ml; *p*<0.001). **(B)** Total reductive abilities of ACL extract and standard butylated hydroxytoluene (BHT). The absorbance (A_700_) was plotted against concentration of sample; higher absorbance value signified greater reducing capacity. **(C)** Hydroxyl radical scavenging capacities of ACL extract and standard mannitol (IC_50_ value: ACL = 121.20±1.22μg/ml and mannitol = 589.06±46.57μg/ml; *p*<0.01). **(D)** Superoxide radical scavenging activities of ACL extract and standard quercetin (IC_50_ value: ACL = 131.900±4.40μg/ml and quercetin = 63.93±4.16μg/ml; *p*<0.01). [Each value represents mean ±SD (n = 6); Where, α = *p*<0.001 Vs 0 μg/ml].

**Fig 2:**
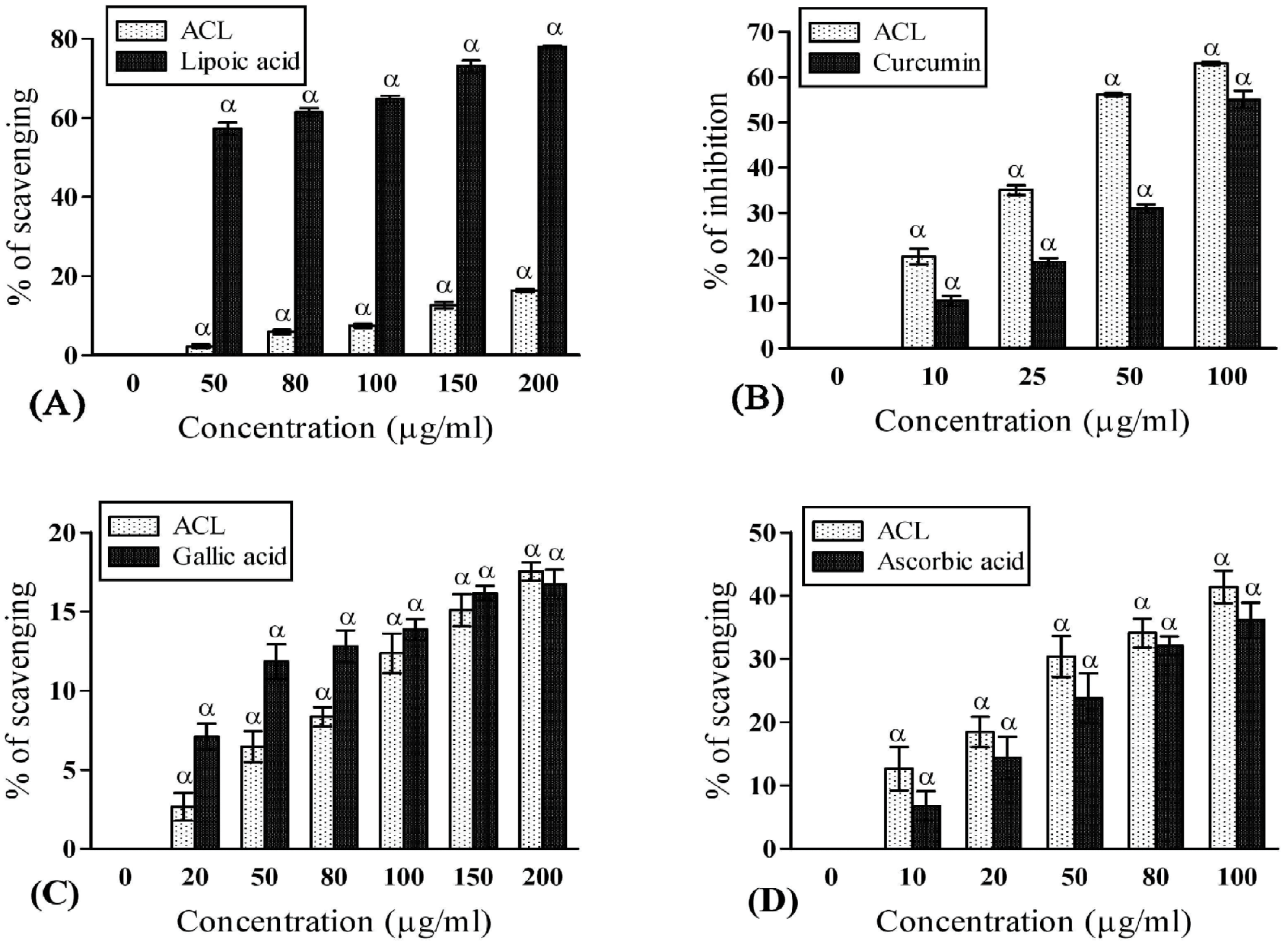
Free-radical scavenging potentials of ACL extract. **(A)** Singlet oxygen scavenging capacities of ACL extract and standard lipoic acid (IC_50_ value: ACL = 1103.79±24.69μg/ml and lipoic acid = 48.40±2.02μg/ml; *p*<0.001). **(B)** Nitric oxide (NO) scavenging activities of ACL extract and standard Curcumin (IC_50_ value: ACL = 45.57±1.33μg/ml and curcumin = 96.88±5.09μg/ml; *p*<0.01). **(C)** Peroxynitrite scavenging activities of ACL extract and standard gallic acid (IC_50_ value: ACL = 854.05±59.96 μg/ml and gallic acid = 734.81±28.30 μg/ml; *p*>0.05). **(D)** Hypochlorous acid (HOCL) scavenging activities of ACL extract and standard ascorbic acid (IC_50_ value: ACL = 130.675±4.78 μg/ml and ascorbic acid = 165.91±16.31μg/ml; *p*<0.01). [Each value represents mean ±SD (n = 6); Where, α = *p*<0.001 Vs 0 μg/ml].

In the present antioxidant profiling, ACL extract exhibited higher free radical scavenging activity (86.30±0.18 at 100μg/ml) than the respective standard (ascorbic acid) as per DPPH assay (Fig 1A). This was evident from the discoloration of DPPH and low IC_50_ value of ACL extract (15.52±0.46μg/ml). Our result proved that the DPPH scavenging activity in ACL was higher or same in comparison to many other recognized medicinal plants [36]. The elevated radical scavenging activity in ACL extract was probably due to the presence of its electron or hydrogen donating capacity [3]. *A*. *catechu* extract has higher reducing capacity than the standard BHT indicating its superior protective ability (Fig 1B). The formation of extremely reactive hydroxyl radical (OH^−^) by way of Haber-Weiss and Fenton reaction in the presence of hydrogen peroxide and excess iron leads to apoptotic cell death through some intermediate pathways [37]. Free hydroxyl radicals are also known for its ability of damaging purine and pyrimidine bases and also affect the deoxyribose backbone [37]. Our experiment with ACL showed that it can significantly scavenge hydroxyl radicals in dose dependant manner (54.91±1.76 at 200 μg/ml) than the mannitol standard (Fig 1C) and many other species [38, 39]. Mitochondria are endogenous reservoir of ROS due to its high O_2_ consumption rate and any dysfunction in mitochondria may result several neurodegenerative diseases [7]. The highly toxic superoxide anion (O^•2-^) originated in mitochondria undergoes spontaneous dismutation generating singlet oxygen (_1_O^2^) which is one of the primary causative agent of lipid peroxidation. Present study exhibited significant O^2-^ (Fig 1D) and _1_O^2^ (Fig 2A) scavenging capacity ultimately providing protection to cellular lipid components by prevention of peroxide formation which is beneficial to the brain function in persons with psychiatric disorders such as AD and PD [1]. Nitric oxide (NO) is a potent mediator of pro-inflammatory cellular activation resulting subsequent inflammatory cellular injury. Moreover, spontaneous coupling of NO with superoxide radicals give rise to highly reactive peroxynitrite (ONOO^-^), which is responsible for causing inflammation in cognitive disorders [40]. Besides, hypochlorous acid (HOCl) is produced at the sites of inflammation due to oxidation of Clˉ ions by the neutrophil enzyme, myeloperoxidase and induces target cell lysis [41]. ACL extract not only possess higher capacity to scavenge NO, ONOO^-^ and HOCl, the quenching activities (63.04±0.37 at 100 μg/ml; 17.52±0.56 at 200 μg/ml; 41.37±2.56 at 100 μg/ml respectively) are also better (Fig 2B–2D) than the respective standards such as curcumin, gallic acid and ascorbic acid. Therefore, ACL extract might help in prevention of inflammation.

**Fig 3:**
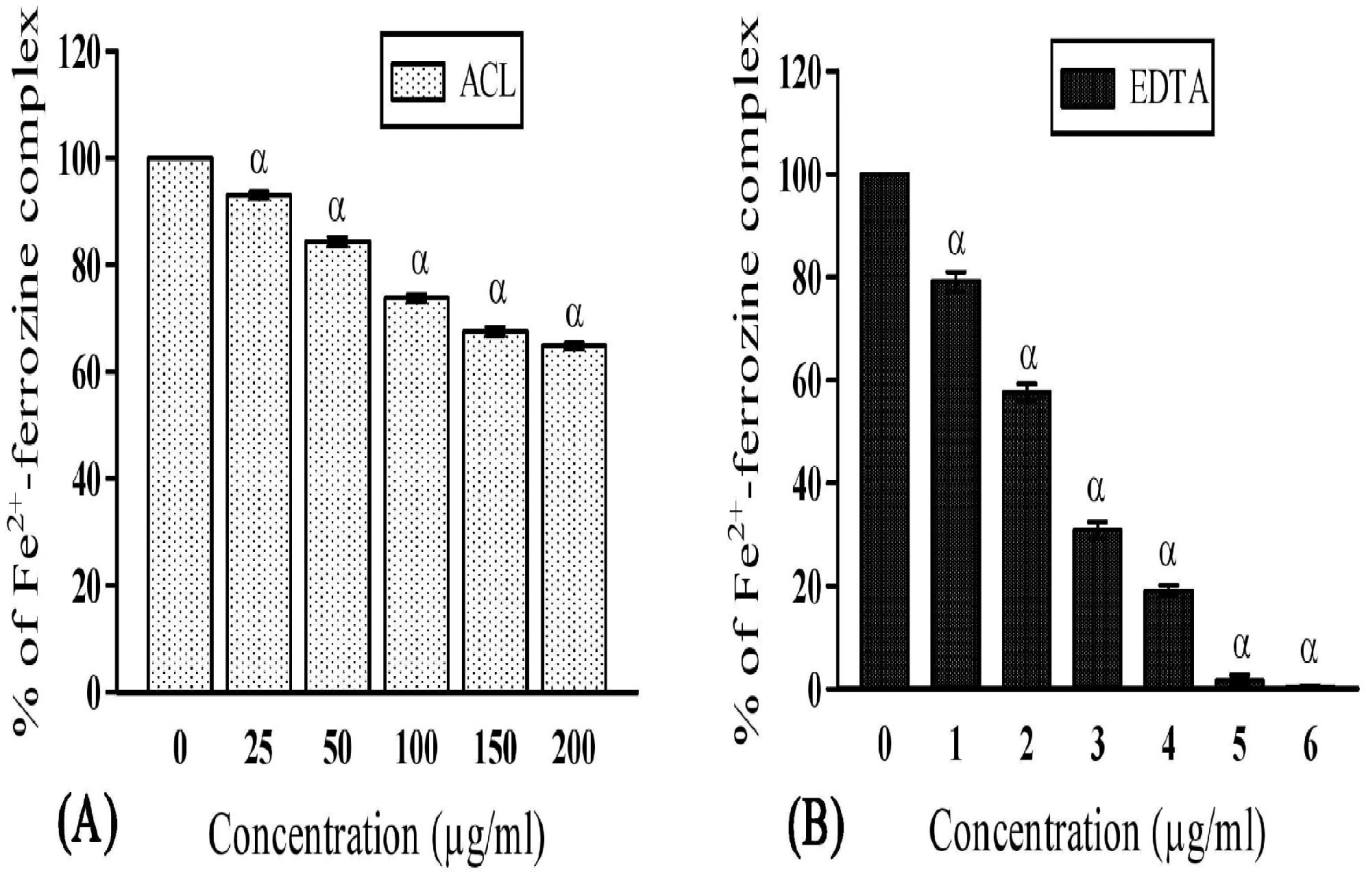
Iron (Fe^2+^)-chelation activities of ACL extract and the reference compound. **(A)** ACL extract and **(B)** standard Ethylenediaminetetraacetic acid (EDTA), represented as % of Fe^2+^-ferrozine complex (IC_50_ value: ACL = 320.63±10.82μg/ml and EDTA = 1.45±0.01μg/ml; p<0.001). [Each value represents mean ±SD (n = 6); Where, α = *p*<0.001 Vs 0 μg/ml].

**Fig 4:**
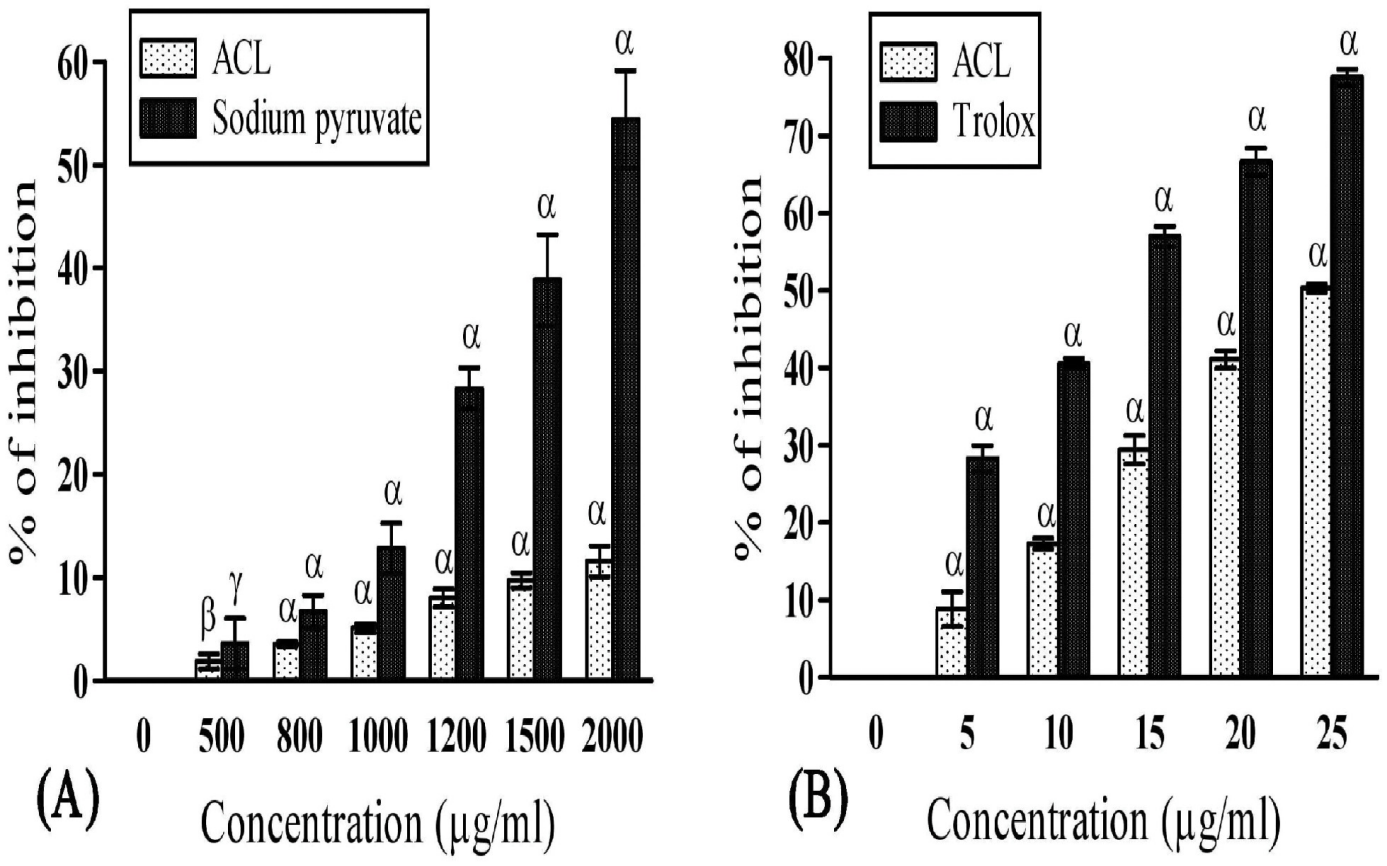
Hydrogen peroxide scavenging and lipid peroxidation inhibitory activity of ACL extract and the reference compound. **(A)** Hydrogen peroxide (H_2_O_2_) scavenging activities of ACL extract and standard sodium pyruvate (IC_50_ value: ACL = 15604.93±613.81μg/ml and sodium pyruvate = 3176.40±140.22μg/ml; *p*<0.001). **(B)** Inhibition of lipid peroxidation by ACL extract and standard trolox (IC_50_ value: ACL = 32.13±0.99μg/ml and trolox = 11.11±0.22μg/ml; *p*<0.001). [Each value represents mean ±SD (n = 6); Where, α = *p*<0.001, β = *p*<0.01 and γ = *p*<0.05 Vs 0 μg/ml.].

Free iron is a potential enhancer of ROS formation as it leads to reduction of H_2_O_2_ and generation of the highly aggressive hydroxyl radical. In the present study, ACL was found to fade the color of ferrozine-complex, indicating its iron chelating activity due to presence of certain components. Moreover, the iron chelating activity (35.14±0.55 at 200 μg/ml) of ACL was found to be higher (Fig 3A and 3B) than many other studied plants [42, 43]. However, the hydrogen peroxide scavenging activity of plant extract was lower (Fig 4A) than the standard (sodium pyruvate), it is still enough to establish its positive role in protecting our body. Lipid peroxidation is a natural metabolic process under normal aerobic conditions and it is one of the most investigated consequences of ROS action [44]. As mentioned earlier, hydroxyl radical and singlet oxygen are the main causative agents of this peroxidation. In presence of antioxidants, lipid peroxidation becomes minimal [44]. Our study revealed significant lipid peroxidation inhibitory activity (Fig 4B). However, the quenching activity was found to be lower (50.28±0.51 at 25 μg/ml) than the respective standard Trolox (77.58±1.0 at 25 μg/ml). Nevertheless, it is still enough to induce a protective effect to establish its positive role in oxidative degradation. The phenolic compounds having redox potentiality play an important role in absorbing and neutralizing the free radicals, scavenging singlet and triplet oxygen, or decomposing peroxidase [45]. Similarly, flavonoids have also been reported to be responsible for its antioxidant activity, as they act on enzymes and pathways involved in anti-inflammatory processes [45]. In the present study, ACL showed decent amount of flavonoid and phenol content exhibiting 13.92±1.60 mg quercetin equivalent per 100 mg of plant extract and 89.59±2.71 mg gallic acid equivalent per 100 mg of plant extract respectively. These contents are the major determinant for such antioxidant activities. Therefore, *A*. *catechu* can well be considered as a plant of medicinal importance and the leaf extract of AC might play a central role in prevention of various neurodegenerative disorders in terms of oxidative stress and free radical generation [46].

Since a large number of evidence exhibit that oxidative stress is closely involved in age-related neurodegenerative diseases, there have been a great number of studies which have examined the positive benefits of antioxidants to reduce or to block neuronal death occurring in the pathophysiology of these disorders. We therefore wanted to perceive whether the ACL extract has any acetylcholinesterase inhibitory activity over CDs. Acetylcholinesterase enzyme hydrolyses the substrate (we used acetylthiocholine iodide) and produces thiocholine which in turn reacts with Ellamn’s reagent (5, 5'-dithiobis-2-nitrobenzoic acid) and 5-thio-2-nitrobenzoic acid is thus produced which is a yellow color compound [47]. The inhibition of AChE enzyme activity is evident by fading the yellow color of the product [47]. Our result exhibited better AChE enzyme inhibition activity (73.47±0.303 at 200μg/ml) with low IC_50_ value of 75.91±2.28μg/ml (IC_50_ of standard eserine = 0.023±0.0005 μg/ml) compared to other medicinal plants including *Andrographis paniculata*, *Cetella asiatica*, *Nelumbo nucifera*, *Nardostachys jatamansi*, *Myristica fragrans* [8,48] suggesting ACL extract as an effective cholinesterase inhibitor for the first time and beneficial against several neurodegenerative disorders or CDs such as dementia, AD, PD.

**Fig 5:**
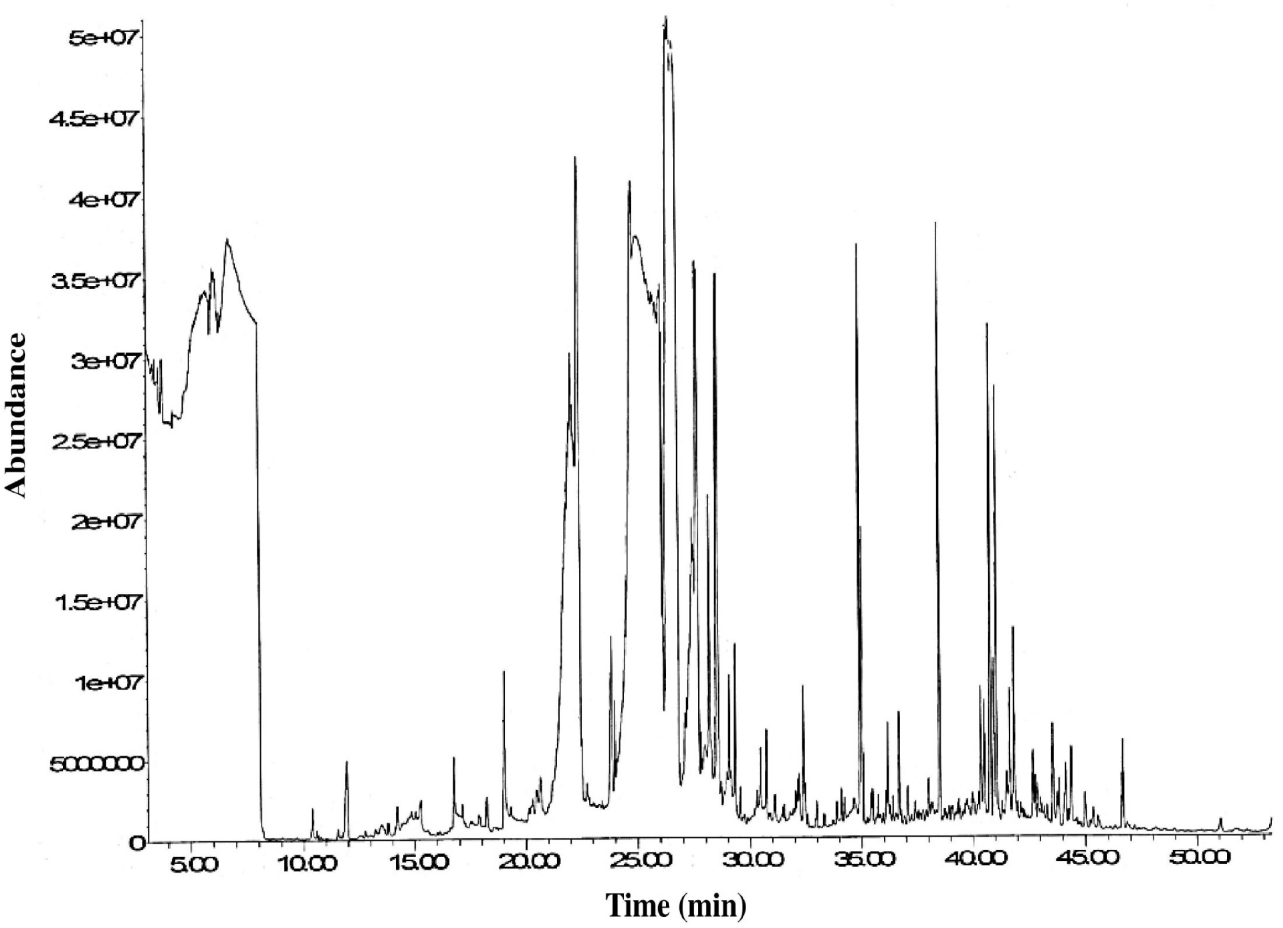
Gas chromatogram-Mass spectroscopy of *A*. *catechu* leaf extract.

**Fig 6:**
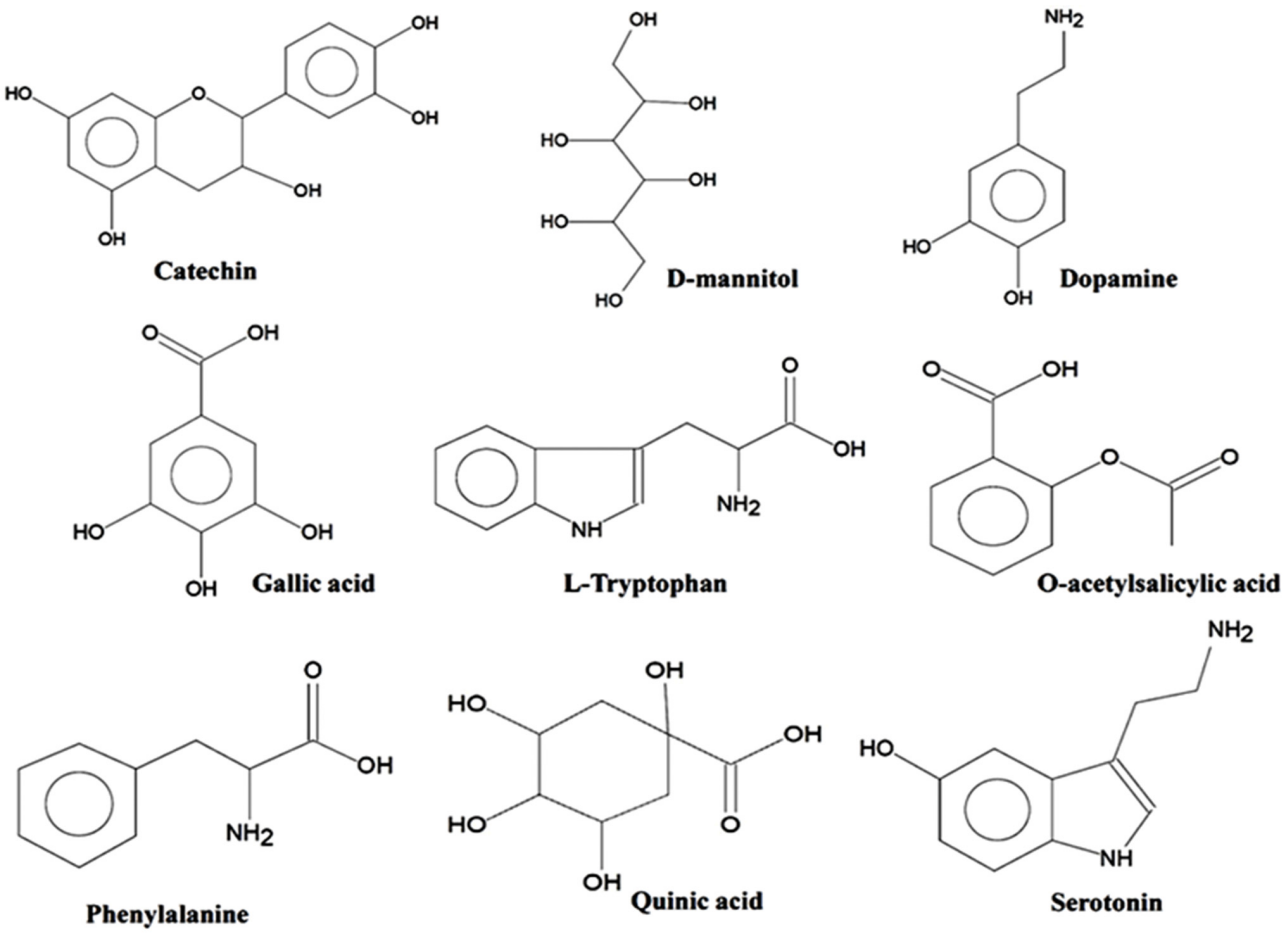
Chemical structures of some essential bioactive metabolites identified in ACL extract by GC-MS.

The enhanced inhibitory effects of ACL against AChE over other medicinal plants prompted us to study the probable compounds present in AC leaf. We have chosen GC-MS analysis and NMR spectroscopy in this regard. GC-MS analysis of ACL (Fig 5) was employed to identify the presence of various bioactive compounds and neurotransmitters, if any. A total of 41 different bioactive metabolites (Fig 6) have been identified by GC-MS analysis (Table 1). Out of 41 compounds we found 5 different compounds which have proven as potent antioxidant activities. These are D-mannitol, gallic acid, epicatechin, catechin and isoquercitrin. Amongst these compounds, gallic acid is known as strong antioxidative agent [49] while D-mannitol, catechin, epicatechin and isoquercitrin are good scavenger of hydroxyl, peroxyl, superoxide and DPPH radicals and exhibit remarkable anti-cancer effects [50–52]. Besides, catechin and epicatechin also show monosamine oxidase inhibitory activity which is partly responsible for PD, AD and others cognitive disorders [53].

**Table 1:** List of metabolites detected in *A*. *catechu* leaf extract by GC-MS analysis.

Sl. No.	Name of the Metabolites	Log of RRR*	
		Average	SD
1.	L-(+) lactic acid	1.07	0.32
2.	L- alanine	0.55	0.19
3.	L- valine	0.94	0.12
4.	Urea	0.23	0.33
5.	Pipecolic acid	1.28	0.42
6.	Glycerol	2.32	0.02
7.	Phosphoric acid	1.30	0.02
8.	L-threonine	1.10	0.17
9.	Glycine	0.56	0.09
10.	Succinic acid	0.77	0.19
11.	Glyceric acid	1.22	0.09
12.	Beta-alanine	0.90	0.10
13.	D- malic acid	1.53	0.03
14.	O-acetylsalisylic acid	0.40	0.21
15.	L- glutamic acid 3 (dehydrated)	1.64	0.13
16.	4-guanidinobutyric acid	1.57	0.25
17.	Phenylalanine	0.59	0.17
18.	Phenylethylamine	1.09	0.08
19.	Meleamic acid	0.48	0.17
20.	L-glutamic acid	0.16	0.05
21.	Lauric acid	1.33	0.50
22.	L- asparagine	1.77	0.05
23.	Xylitol	2.14	0.25
24.	Arabitol	0.79	0.05
25.	Putrescine	1.04	0.09
26.	Methyl-beta-D-galactopyranoside	1.25	0.05
27.	Quinic acid	1.41	0.48
28.	Allantoin	1.69	0.06
29.	Tyramine	3.22	0.11
30.	D-sorbitol	1.19	0.07
31.	D-mannitol	1.13	0.09
32.	Gallic acid	3.19	0.02
33.	Palmitic acid	1.80	0.08
34.	Dopamine (hydroxytyramine)	1.02	0.07
35.	L-tryptophan	1.52	0.02
36.	Stearic acid	1.10	0.22
37.	Serotonin	1.01	0.04
38.	Sucrose	2.28	0.00
39.	(-)- epicatechin	2.14	0.02
40.	Catechin	2.05	0.07
41.	Isoquercitrin	1.69	0.03

*RRR: Relative Response Ratio; SD: Standard Deviation.

**Fig 7:**
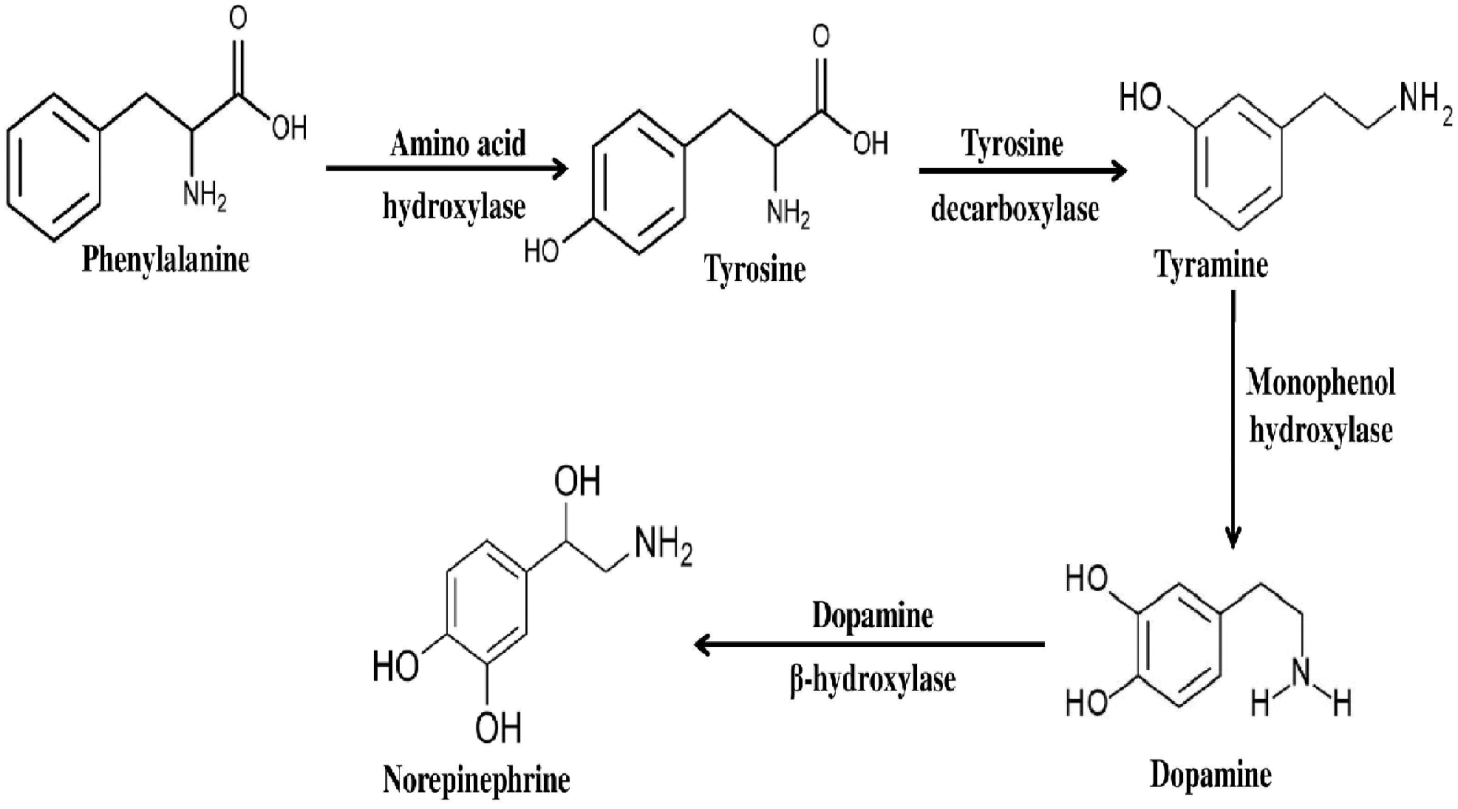
Schematic representation of possible biosynthetic pathway of catecholamines identified in ACL extract.

We have also found a few bioactive compounds which have either anti-neurodegenerative properties or are known as precursor of compounds which are anti-CDs. These compounds were found to be tyramine, dopamine, and serotonin. Out of these bio-active compounds, dopamine and norepinephrine are collectively called catecholamine [54]. Generally, the catecholamines are synthesised following two equally active routes [55]. In one of the routes, phenylalanine converts into tyrosine with the help of amino acid hydroxylase. Tyrosine then decarboxylated to tyramine using tyrosine decarboxylase and subsequently produces dopamine and norepinephrine (noradrenaline) catalysed by enzyme monophenol hydroxylase and dopamine beta- hydroxylase respectively. In our present study, the GC-MS analysis of ACL extract revealed the presence of neurotransmitters like, phenylalanine, tyramine, dopamine, serotonin etc. Therefore, we may conclude that the synthesis of catecholamines in ACL probably follows the described route (Fig 7). In addition, it may conclude that the serotonin probably synthesised from its immediate precursor L-tryptophan. Several studies and reviews [55–57] exhibited that catecholamines and other neurotransmitters have distinct role to combat against cognitive disorders like AD, PD and dementia.

**Fig 8:**
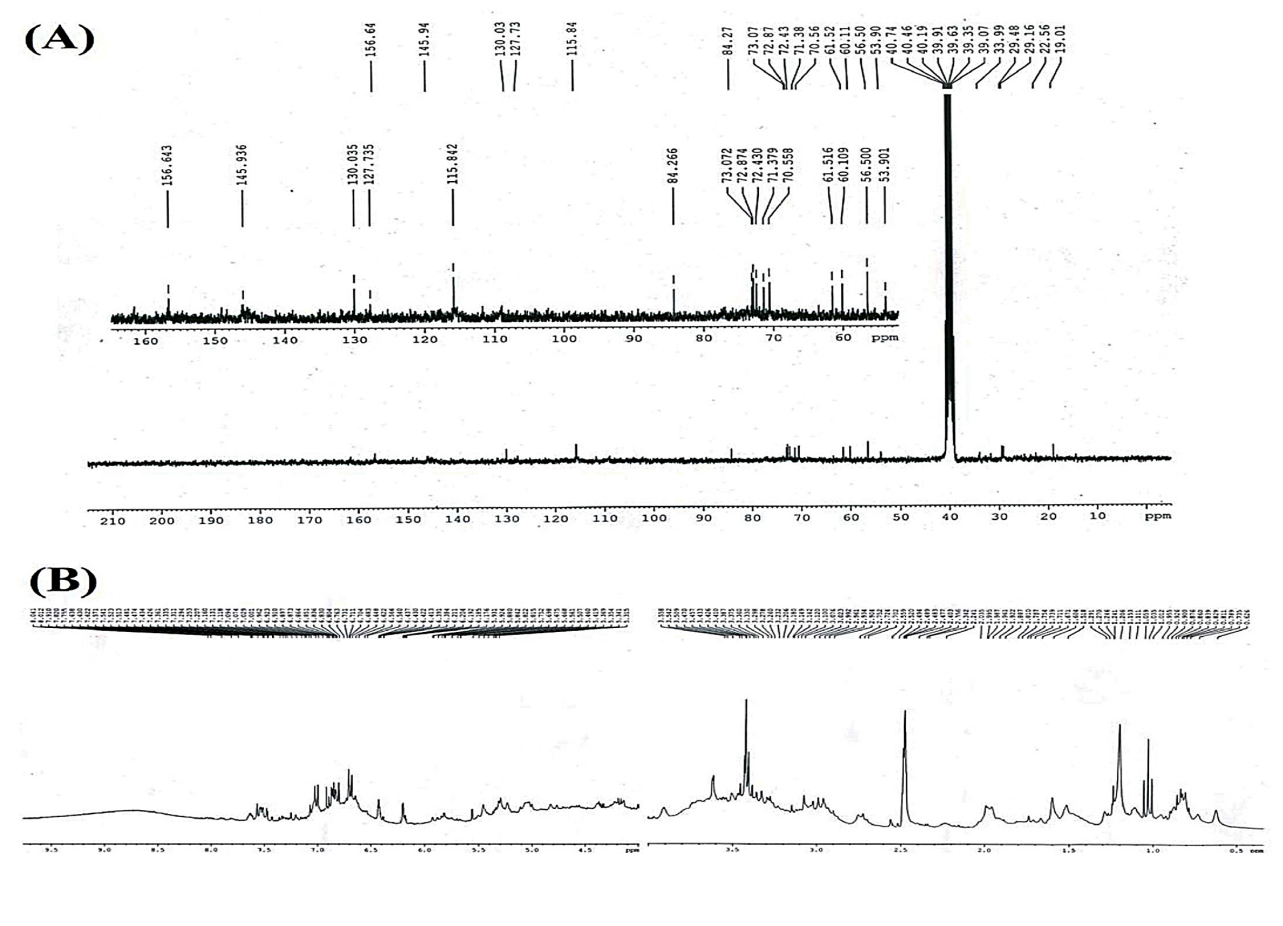
(A) ^13^C NMR spectra and (B) ^1^H NMR spectra of ACL extract.

Furthermore, NMR spectroscopy of ACL extract was employed in the present study. From the ^13^C NMR spectra (Fig 8A), several peaks in the aliphatic (*δ* = 19.0–84.2) and aromatic region (*δ* = 115.8–156.6) have been identified confirming the presence of both aliphatic and aromatic carbons [35]. Similarly, ^1^H NMR spectra (Fig 8B) exhibited peaks related to aliphatic, aromatic and olefinic protons. In addition, a hump near *δ* = 8.75 indicates the presence of amine functional group supporting the result found in GC-MS analysis which might be responsible for the bioactive properties of the ACL extract.

**Fig 9:**
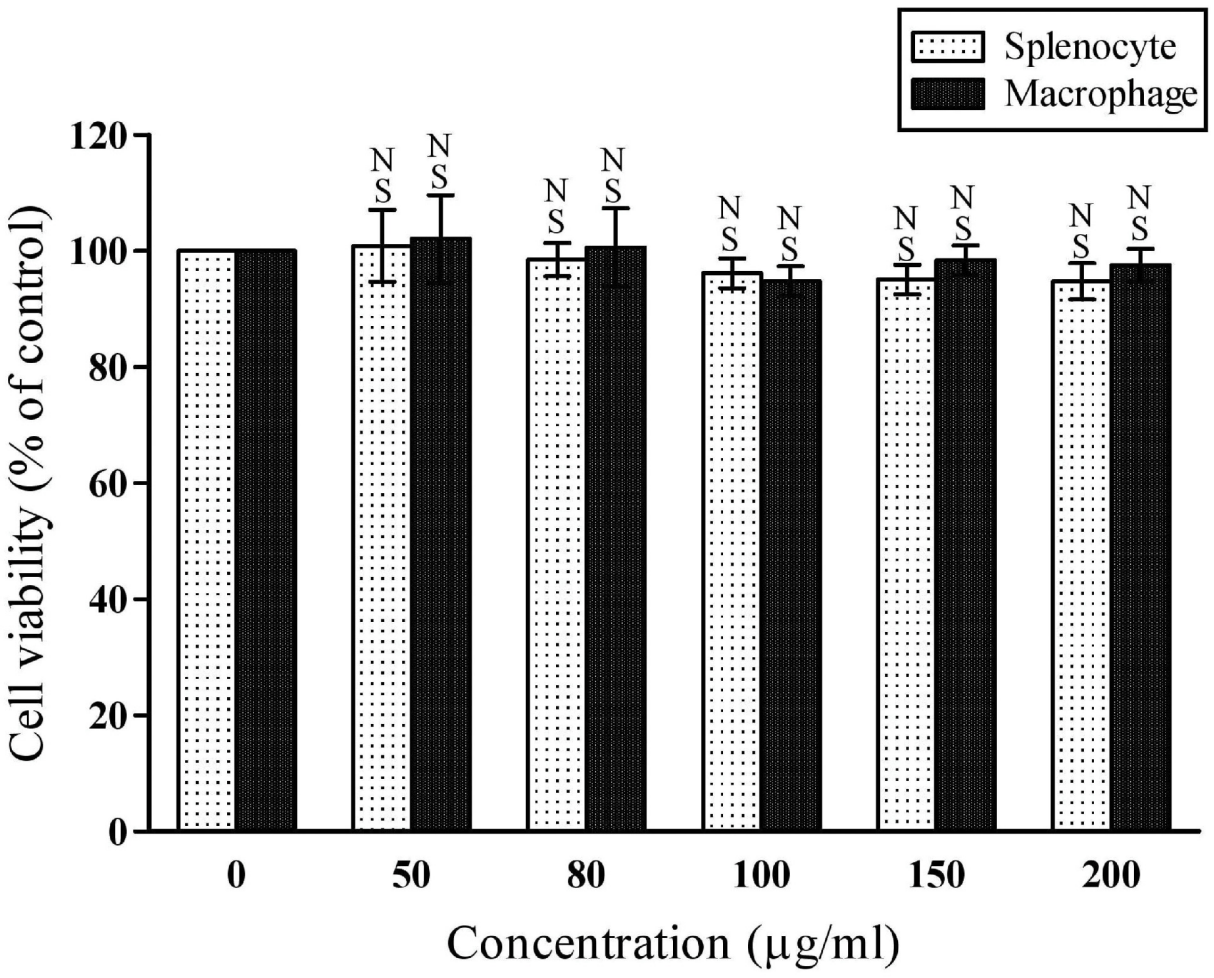
The effect of ACL extract on the viability of murine splenocytes and peritonealexudate macrophages, evaluated by MTT method. Each value represents mean ±SD (n = 6); Where, ^NS^*p* = 0>0.05.

The cytotoxic effect of ACL was further assessed on mice splenocyte and macrophage cells whether the extract has any deleterious effects on normal body cells. The effect of the ACL on the cell viability was non-significant (*P*>0.05) relative to control. At highest dose (200 μg/ml) of extract, the magnitude of cell viability compared to the control was 94.77±3.09% and 97.48±2.85% for cultured splenocytes and macrophages respectively (Fig 9) displaying no cytotoxic effect on either of splenocyte or macrophage cells. Hence, ACL extract represents as safety stuff to consume.

## Conclusion

The occurrence of dementia along with other cognitive disorder is increasing. Survey exhibited that near about 35.6 million people lived with dementia worldwide in 2010 and the number is expected to be doubled in the next 20 years [58]. However, the prevalence of dementia is more in North and South America and Europe [58–60] in comparison to Sub-Saharan Africa and India [61]. Besides genetic factors, relatively low occurrence of dementia in India may be attributed to socio-cultural activities, lifestyle, dietary habits etc [62]. From the lifestyle and dietary point of view, one of the major differences between the population of Indian sub-continent and rest of the world is the chewing of betel leaf (*Piper betel*) and betel nut (*Areca catechu*) along with lime and Khair (*A*. *catechu*). Several workers [63,64] have been studied the medicinal properties of betel leaf. In a recent study, Sullivanet al. [65] showed that betel nut chewing is positively associated with less severe symptoms of schizophrenia.

We, therefore, wanted to perceive whether *Acacia catechu* leaf has effect over CDs (beneficial or harmful). Result exhibited that ACL extract had significant antioxidant activity against various free radicals. The pronounced antioxidant activity of ACL extract was found to be potent due to its phenolic and flavonoid content. Presence of dopamine in ACL extract made it more significant and potential for use in the preparation of anti-CD drugs. Moreover, ACL extract was found to be nontoxic and safer through MTT cell viability assay. The chemical characterization of ACL using NMR and GC-MS revealed presence of several antioxidative compounds with potential benefit in psychiatric disorders such as AD and PD. However, extensive research with human models with those bioactive compounds, found in ACL will justify its use in novel drug delivery systems.
